# Magnetic-field sensor with self-reference characteristic based on a magnetic fluid and independent plasmonic dual resonances

**DOI:** 10.3762/bjnano.10.23

**Published:** 2019-01-22

**Authors:** Kun Ren, Xiaobin Ren, Yumeng He, Qun Han

**Affiliations:** 1 College of Precision Instrument and Opto-electronics Engineering,Tianjin University; Key Laboratory of Opto-electronics Information Technology, Ministry of Education, Tianjin 300072, China; 2 School of Science, Tianjin University of Science and Technology, Tianjin 300222, China

**Keywords:** dual resonance, magnetic fluid, magnetic sensor, plasmonic waveguide, self-reference, surface plasmon polaritons

## Abstract

A magnetic-field sensor with self-reference characteristic based on metal–dielectric–metal (MDM) plasmonic waveguides and a magnetic fluid (MF) is proposed and theoretically investigated. Independent dual resonances are supported by the coupled resonator–waveguide system. The physical mechanisms of dual resonances are analyzed by the temporal coupled-mode theory. The transmission response to an external magnetic field is explored by using the remarkable tunability of the refractive index of the MF. Based on the different dependence of two resonances on the external field, a magnetic-field sensor with self-reference characteristic is achieved. The magnetic-field nanosensor shows an excellent performance with a high sensitivity of 27 pm/Oe, i.e., 270 pm/mT. The proposed sensor takes advantage of the refractive-index tunability of the MF and the compactness of the MDM waveguide structure. This research may open new opportunities to design nanoscale magnetic sensors with good performance.

## Introduction

Sensors that can detect the change of environmental conditions are one of the most important devices in optical communication. Optical sensors are obtained by monitoring the change of optical properties based on, e.g., thermo-optic, electro-optic, and magneto-optic effects. In the area of magnetic-field sensors, magnetic fluids (MFs) or ferrofluids have attracted a lot of research interest in recent years [1].

A MF is a stable colloidal suspension of ferromagnetic nanoparticles in certain suitable liquid carriers. It has the remarkable property that the refractive index can be tuned in an applied magnetic field [2–3]. The tunability results from the field-induced structural reorganization of suspended magnetic colloidal particles [4]. This field-dependent property of a MF has been utilized to measure magnetic fields and current intensities [5–6]. Other applications of MFs in optical devices have been proposed, such as tunable optical filters [7], optical switches [8], modulators [9] and optical sensors [10]. In particular, optical-fiber magnetic sensors have been developed by combining MFs with optical-fiber technology [11–15]. Optical-fiber magnetic-field sensors have the advantages of easy fabrication and compactness.

In recent years, compact optical devices based on surface plasmon polaritons (SPPs) have been reported. SPPs propagate along the dielectric–metal interface with the amplitudes decaying exponentially into both sides [16]. The deep subwavelength confinement of SPPs leads to the development of various integrated photonic components, such as filters [17], modulators [18], interferometers [19], optical switches [20] and nanosensors [21–22]. As important plasmonic structures, metal–dielectric–metal (MDM) waveguides have attracted considerable attention. Various kinds of plasmonic devices containing MDM waveguides have been investigated [23–27]. MDM waveguide–cavity coupled systems have been reported such as stub cavities [24], side-coupled rectangular cavities [25], T-shape cavities [26] and ring–groove joint cavities [27].

In this paper we propose a compact magnetic-field sensor based on a MF and a plasmonic structure. As far as we know, the combination of a MF with a plasmonic waveguide has not been reported to date. The unique magnetic-optical properties of MFs are the basis of the optical sensor. The plasmonic structure is a MDM waveguide–cavity coupled system that is suitable for chip-scale integration. The proposed sensor takes advantage of the refractive index tunability of MFs as well as of the compactness of the MDM waveguide structure. Furthermore, the proposed magnetic-field sensor has a self-reference characteristic, which can guarantee the detection accuracy. A simple and compact self-reference sensor with high sensitivity is achieved and it is promising in the integrated sensing and detection of magnetic fields.

## Methods

**Figure 1:**
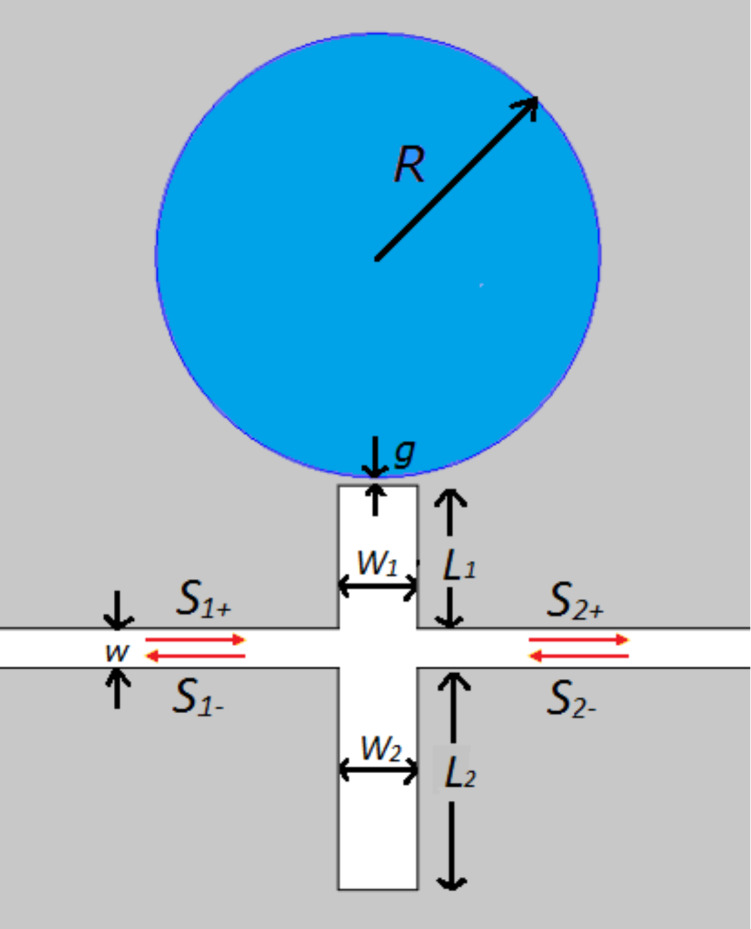
Schematic diagram of the MDM waveguide–resonator structure. The width of the waveguide is *W* and the radius of the disk is *R*. *L*_1_, *L*_2_, and *W*_1_, *W*_2_ denote the length and the width of two stubs, respectively. The coupling distance between the disk resonator and the upper stub is *g*.

The proposed plasmonic nanostructure is schematically shown in Figure 1. Two stubs are located on each side of the MDM waveguide. A disk resonator is coupled to the upper stub with a coupling distance *g*. *L*_1,_ *L*_2_, and *W*_1_, *W*_2_ denote the length and the width of the stubs*,* respectively. The radius of disk is *R* and the width of the MDM waveguide is *W*. The dielectric in the stub and waveguide is water. The disk resonator is filled with magnetic fluid. The background metal in the grey part is silver, the complex permittivity of which is characterized by the well-known Drude model: 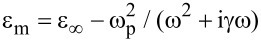, where ε_∞_ = 3.7 is the permittivity at infinite angular frequency, the bulk plasma frequency is ω_p_ = 1.38 × 10^16^ Hz, the damping frequency of the oscillations is γ = 2.73 × 10^13^ Hz, and ω is the angular frequency of the incident light.

The effective refractive index for SPPs is given by *n*_eff_ = β/*k*_0_. The propagation constant β can be obtained from the dispersion equation [28]: *ε*_d_*k*_m_ + *ε*_m_*k*_d_ tanh (*k*_d_*w*/2) = 0, where *k*_d_ and *k*_m_ are the transverse propagation constants in the dielectric and the metal, respectively:


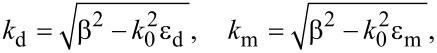


where *k*_0_ represents the wave number of light in free space. The temporal coupled-mode theory (CMT) is used to account for the dynamic transmission characteristics. For the resonator-coupled waveguide system in Figure 1, the coupling coefficient between waveguide and stub 1 (stub 2) are κ_w1_ (κ_w2_). The coupling coefficient between disk resonator and stub 1 is denoted by κ_1d_. The decay rates due to the internal loss in the cavities are κ_1_, κ_2_ and *κ*_d_, which can be neglected. The amplitudes of the incoming and outgoing waves in in waveguide are denoted by *S_i_*_+_ and *S_i_*_−_ (*i* = 1,2).

Considering a waveguide structure which is composed of a stub coupled with a disk cavity, the normalized amplitude *a*_1_ of the stub and *a*_d_ of the disk can be expressed as follows:

[1] $$\begin{array}{c} \frac{\text{d}a_{1}}{\text{d}t}=\left(j\omega_{1}-\kappa_{\text{w1}}-\kappa_{\text{1d}}-\kappa_{1}\right)a_{1}\\ +j\sqrt{\kappa_{\text{w1}}}S_{1+}+j\sqrt{\kappa_{\text{w1}}}S_{2+}+j\sqrt{\kappa_{\text{1d}}}a_{\text{d}}, \end{array}$$

[2] $$\frac{\text{d}a_{\text{d}}}{\text{d}t}=\left(j\omega_{\text{d}}-\kappa_{\text{1d}}\right)a_{\text{d}}+j\sqrt{\kappa_{\text{1d}}}.$$

According to energy conservation, the amplitude of the incoming and the outgoing waves in coupled waveguides should satisfy the following relationships:

[3] $$S_{2+}=S_{1+}+j\sqrt{\kappa_{\text{w1}}}a_{1},$$

[4] $$S_{1-}=S_{2-}+j\sqrt{\kappa_{\text{w1}}}a_{1}.$$

When an optical wave with frequency ω is launched only from the input port of the bus waveguide, *S*_2+_ = 0. The transmission and reflection coefficients of the stub–disk system can be derived as:

[5] $$\begin{array}{c} t_{\text{1d}}=\frac{S_{2-}}{S_{1+}}\\ \;=\frac{j\left(\omega -\omega_{1}\right)+\kappa_{\text{1d}}+\kappa_{1}+\frac{\kappa_{\text{1d}}}{j\left(\text{ω}-{\text{ω}}_{\text{d}}\right)+\kappa_{\text{1d}}}}{j\left(\omega -\omega_{1}\right)+\kappa_{\text{w1}}+\kappa_{\text{1d}}+\kappa_{1}+\frac{\kappa_{\text{1d}}}{j\left(\omega -\omega_{\text{d}}\right)+\kappa_{\text{1d}}}}\\ =1-\frac{\kappa_{w1}}{\beta_{1}+\frac{\kappa_{1d}}{\beta_{d}}}, \end{array}$$

[6] $$\begin{array}{c} r_{\text{1d}}=\frac{S_{1-}}{S_{1+}}\;\\ =-\frac{\kappa_{\text{w1}}}{j\left(\omega -\omega_{1}\right)+\kappa_{\text{w1}}+\kappa_{\text{1d}}+\frac{\kappa_{\text{1d}}}{j\left(\omega -\omega_{\text{1d}}\right)+\kappa_{\text{1d}}}}\\ =-\frac{\kappa_{\text{w1}}}{\beta_{1}+\frac{\kappa_{\text{1d}}}{\beta_{\text{d}}}} \end{array}$$

where β_1_ = *j*(ω − ω_1_) + κ_w1_ + κ_1d_ + κ_1_, and β_d_ = *j*(ω − ω_1d_) + κ_1d_. From Equation 5, the transmission efficiency of stub–disk coupled system can be expressed as:

[7] $$T={\left|t_{\text{1d}}\right|}^{2}={\left|1-\frac{\kappa_{\text{w1}}}{\beta_{1}+\frac{\kappa_{\text{1d}}}{\beta_{\text{d}}}}\right|}^{2}.$$

Neglecting the internal loss κ_1_, we find the transmission response of stub–disk system to be

[8] $$T={\left|t_{\text{1d}}\right|}^{2}={\left|\frac{j\left(\omega -\omega_{1}\right)+\kappa_{\text{1d}}+\frac{\kappa_{\text{1d}}}{j\left(\text{ω}-{\text{ω}}_{\text{d}}\right)+\kappa_{\text{1d}}}}{j\left(\omega -\omega_{1}\right)+\kappa_{\text{w1}}+\kappa_{\text{1d}}+\frac{\kappa_{\text{1d}}}{j\left(\omega -\omega_{\text{d}}\right)+\kappa_{\text{1d}}}}\right|}^{2}.$$

If there is no disk resonator coupled to the stub, which means there is only one stub resonator, then κ_1d_ = 0, and Equation 5 and Equation 6 are simplified. Transmission and reflection coefficients of a waveguide with one stub are written as:

[9] $$t_{i}=\frac{j\left(\omega -\omega_{i}\right)+\kappa_{i}}{j\left(\omega -\omega_{i}\right)+\kappa_{\text{w1}}+\kappa_{i}}=1-\frac{\kappa_{\text{w}i}}{\beta_{i}},$$

[10] $$r_{i}=-\frac{\kappa_{\text{w}i}}{j\left(\omega -\omega_{i}\right)+\kappa_{\text{w}i}+\kappa_{i}}\;=-\frac{\kappa_{\text{w}i}}{\beta_{i}}.$$

The subscripts *i* = 1, 2 stand for stub 1 and stub 2, respectively; β_2_ = *j*(ω − ω_2_) + κ_w2_ + κ_2_. Through the use of Equation 9, the transmission efficiency of an individual resonator stub is given by:

[11] $$T={\left|t_{i}\right|}^{2}={\left|1-\frac{\kappa_{\text{w}i}}{\beta_{i}}\right|}^{2}=\frac{{\left(\omega -\omega_{i}\right)}^{2}+\kappa_{i}^{2}}{{\left(\omega -\omega_{i}\right)}^{2}+{\left(\kappa_{\text{w}i}+\kappa_{i}\right)}^{2}}.$$

Equation 11 holds for any individually coupled resonator whether it is a stub or a disk resonator. Therefore Equation 11 presents the general expression of transmission response for an individual resonator. In this paper, the subscripts *i* = 1, 2, d stand for stub 1, stub 2 and the disk resonator, respectively. Equation 11 is consistent with the result in [29].

For a waveguide system coupled to multiple resonators, the incident and output waves through the entire system show a transfer characteristic. The transmission is determined by a transfer matrix [30]. We regard that the structure shown in Figure 1 is composed of two resonator subsystems. The first subsystem consists of stub 1 and disk, and the second subsystem is only stub 2. Then the transmission of the entire system is described by:

[12] $$T={\left|\frac{t_{\text{1d}}t_{2}}{1-r_{\text{1d}}r_{2}e^{j\psi }}\right|}^{2},$$

where *t*_1d_ (*r*_1d_) and *t*_2_ (*r*_2_) denote the transmission (reflection) coefficients of 1st and 2nd resonator subsystem. ψ represents the phase difference between the subsystems. In our case the separation distance between two stubs is zero, ψ = 0, so the transmission is written as:

[13] $$T={\left|\frac{\left(1-\frac{\kappa_{\text{w1}}}{\beta_{1}+\frac{\kappa_{\text{1d}}}{\beta_{\text{d}}}}\right)\left(1-\frac{\kappa_{\text{w2}}}{\beta_{2}}\right)\;}{1-\left(\frac{\kappa_{\text{w1}}}{\beta_{1}+\frac{\kappa_{\text{1d}}}{\beta_{\text{d}}}}\right)\left(\frac{\kappa_{\text{w2}}}{\beta_{2}}\right)}\right|}^{2}.$$

From Equation 13 the transmission of entire system is obtained. The results for simple resonator structures can be derived based on Equation 13. If there is no stub 2, κ_w2_ = 0_,_ then Equation 13 reduces to


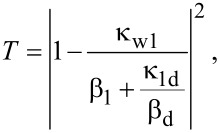


which is the same as Equation 7. It describes the transmission efficiency of the stub–disk coupled system. If there is no disk, κ_1d_ = 0, then Equation 13 is simplified to Equation 14 (see below).

[14] $$T={\left|\frac{\left(1-\frac{\kappa_{\text{w1}}}{\beta_{1}}\right)\left(1-\frac{\kappa_{\text{w2}}}{\beta_{2}}\right)\;}{1-\left(\frac{\kappa_{\text{w1}}}{\beta_{1}}\right)\left(\frac{\kappa_{\text{w2}}}{\beta_{2}}\right)}\right|}^{2}={\left|\frac{\left(1-\frac{\kappa_{\text{w1}}}{j\left(\omega -\omega_{1}\right)+\kappa_{\text{w1}}+\kappa_{\text{1d}}+\kappa_{1}}\right)\left(1-\frac{\kappa_{\text{w2}}}{j\left(\omega -\omega_{2}\right)+\kappa_{\text{w2}}+\kappa_{2}}\right)\;}{1-\left(\frac{\kappa_{\text{w1}}}{j\left(\omega -\omega_{1}\right)+\kappa_{\text{w1}}+\kappa_{\text{1d}}+\kappa_{1}}\right)\left(\frac{\kappa_{\text{w2}}}{j\left(\omega -\omega_{2}\right)+\kappa_{\text{w2}}+\kappa_{2}}\right)}\right|}^{2}.$$

This is the transmission efficiency of a two-side coupled-stubs structure. Assume the two stubs are identical, then κ_w1_ = κ_w2_ = κ_w_, β_1_ = β_2_ = β_0_ and ω_1_ = ω_2_ = ω_0_. Ignoring the loss, Equation 14 can be derived as:


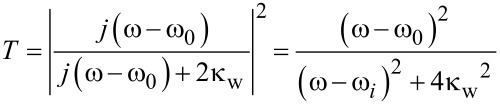


Thus, the transmission efficiency of two-side coupled cavities is obtained, which is consistent with the results in [31]. If there is no disk and no stub 2, κ_1d_ = 0 and κ_w2_ = 0, then Equaiton 13 reduces to


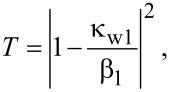


which is same as Equation 11. This is exactly the transmission efficiency of a one-stub system.

## Results and Discussion

Numerical simulations were performed by using COMSOL Multiphysics to investigate the spectral response. In the simulations, we fix *W* = 50 nm and *g* = 10 nm. The radius of the disk is *R* = 280 nm. The widths of stubs are *W*_1_ = 100 nm, and *W*_2_ = 100 nm. The lengths of the stubs are *L*_1_ = 180 nm, and *L*_2_ = 280 nm.

**Figure 2:**
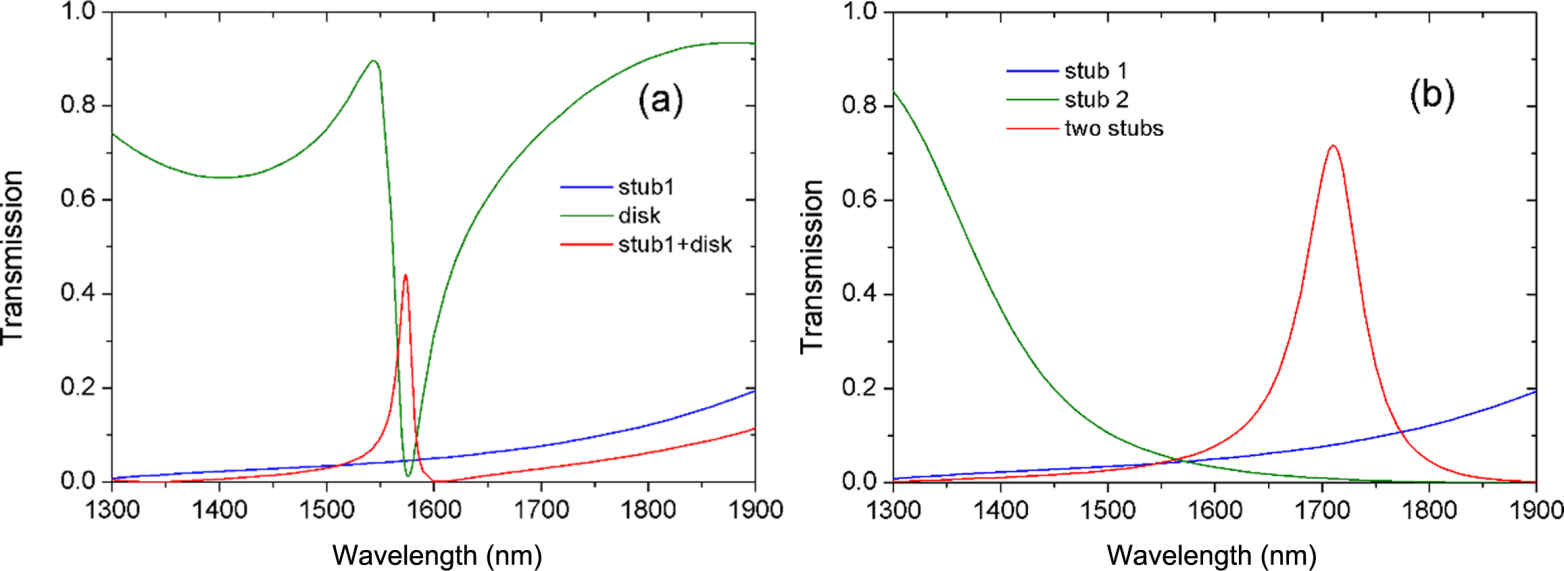
Transmission spectra for different resonator-coupled structures. The radius of the disk is *R* = 280 nm. The lengths of the stubs are *L*_1_ = 180 nm, *L*_2_ = 280 nm. (a) The individual stub 1 resonator, individual disk resonator, and coupled stub–disk system. (b) The individual stub 1 resonator, individual stub 2 resonator, and coupled-stubs system.

The obtained transmission spectra for different resonator-coupled waveguide structures are plotted in Figure 2. It is seen that for the individual resonator, there is a wavelength range in which the transmittance is low, as shown by the green and blue lines. For the stub–disk coupled waveguide structure, a transmission peak appears in the transmission dip, as shown by the red line in Figure 2a. For two-stub coupled structures, a transmission peak is found, as shown by red line in Figure 2b. This phenomenon is similar to electromagnetically induced transparency (EIT) [32–33].

The above transmission results can be quantitatively explained by our theory model. Equation 12 demonstrates that the minimum transmission *T*_min_ occurs when ω*_i_* =ω. Then, *T_i,_*_min_ = |κ*_i_*/(κ_w_*_i_* + κ*_i_*)|^2^. Neglecting the internal loss κ*_i_*, we have *T*_min_ = 0. This means that a wave with resonance frequency ω*_i_* will be suppressed and not transmitted. Based on Equation 11, one can explain the transmission dip around the resonance frequencies ω_1_, ω_2_ and ω_d_ for the individual resonators stub 1 and stub 2, and the disk, respectively.

Note that the individual stub resonator has very broad transmission dip. The broad dip is caused by the direct coupling. Because stub resonators are directly connected to the MDM waveguide, the energy stored in the stubs will be reduced and the quality factor of the stubs will decrease. As a result, broad transmission dips are formed. The disk resonator has narrower transmission dip than the stubs. This is because the disk is side-coupled to the waveguide. Besides, the disk resonator can support whispering gallery modes. These can greatly reduce the propagation loss in the disk resonator. Therefore its quality factor is higher and the corresponding transmission dip is narrower. The spectral shape of the transmission for a single resonator agrees well with the theoretical analysis.

When a disk is coupled to the upper stub 1, cavities modes can directly couple with each other through their evanescent field at small gap distances. When ω_1_ = ω_d_ = ω*,* the transmittance of Equation 8 becomes *T* = |(κ_1d_ + 1)/(κ_w1_ + κ_1d_ + 1)|^2^. This indicates that a transmission peak emerges in the transmission dip. Based on Equation 8 and Equation 11, we can explain the EIT-like phenomenon of the transmission peak in Figure 2.

**Figure 3:**
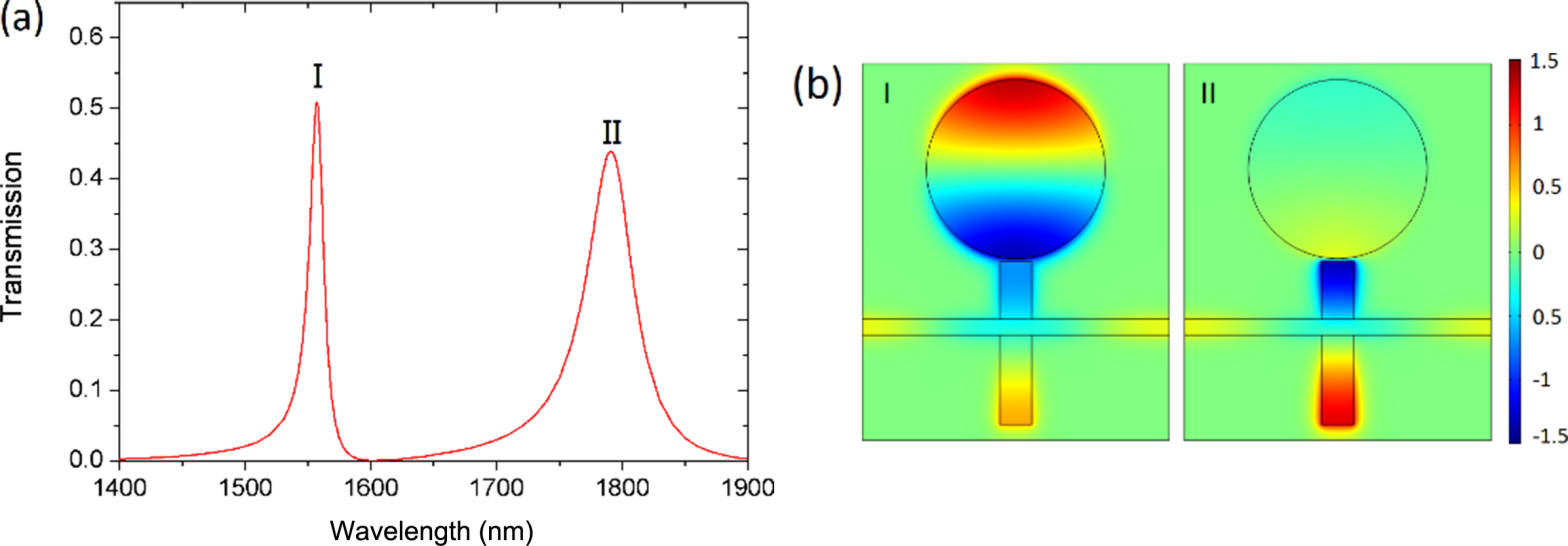
(a) Transmission spectra of the entire structure. (b) *H_z_* field patterns at the peak wavelengths of the two resonances. The radius of the disk is *R* = 280 nm. The lengths of the stubs are *L*_1_ = 180 nm, *L*_2_ = 280 nm.

Figure 3 shows the transmission spectrum for resonator-coupled waveguide structures in which two transmission peaks appear. The corresponding peak wavelengths are λ_I_ = 1557 nm and λ_II_ = 1791 nm. This phenomenon can also be explained by our theory. Equation 13 indicates that there would be a transmission peak at frequency ω_d_ = ω as long as ω_1_ and ω_d_ are close to each other, and another peak at frequency ω_2_ = ω, as long as ω_1_ and ω_2_ are close to each other. According to this analysis, we know there are two transmission peaks. Peak I is near the resonance frequency of the disk (ω_d_), and peak II is around the resonance frequency of stub 2 (ω_2_). The obtained spectra agree well with the theoretical prediction.

To further reveal the origin of the transmission peaks in Figure 3a, we plot the distribution of the magnetic field *H_z_* at the two resonance wavelengths in Figure 3b. It is seen that the energy is mostly confined in the disk resonator at the frequency of resonance peak I. In contrast, at the frequency of resonance peak II the energy is confined in the two stubs and there is almost no energy in the disk resonator. We infer that peak I results from the coupling between disk and stub 1 and peak II is related to the resonance between stub 1 and stub 2.

**Figure 4:**
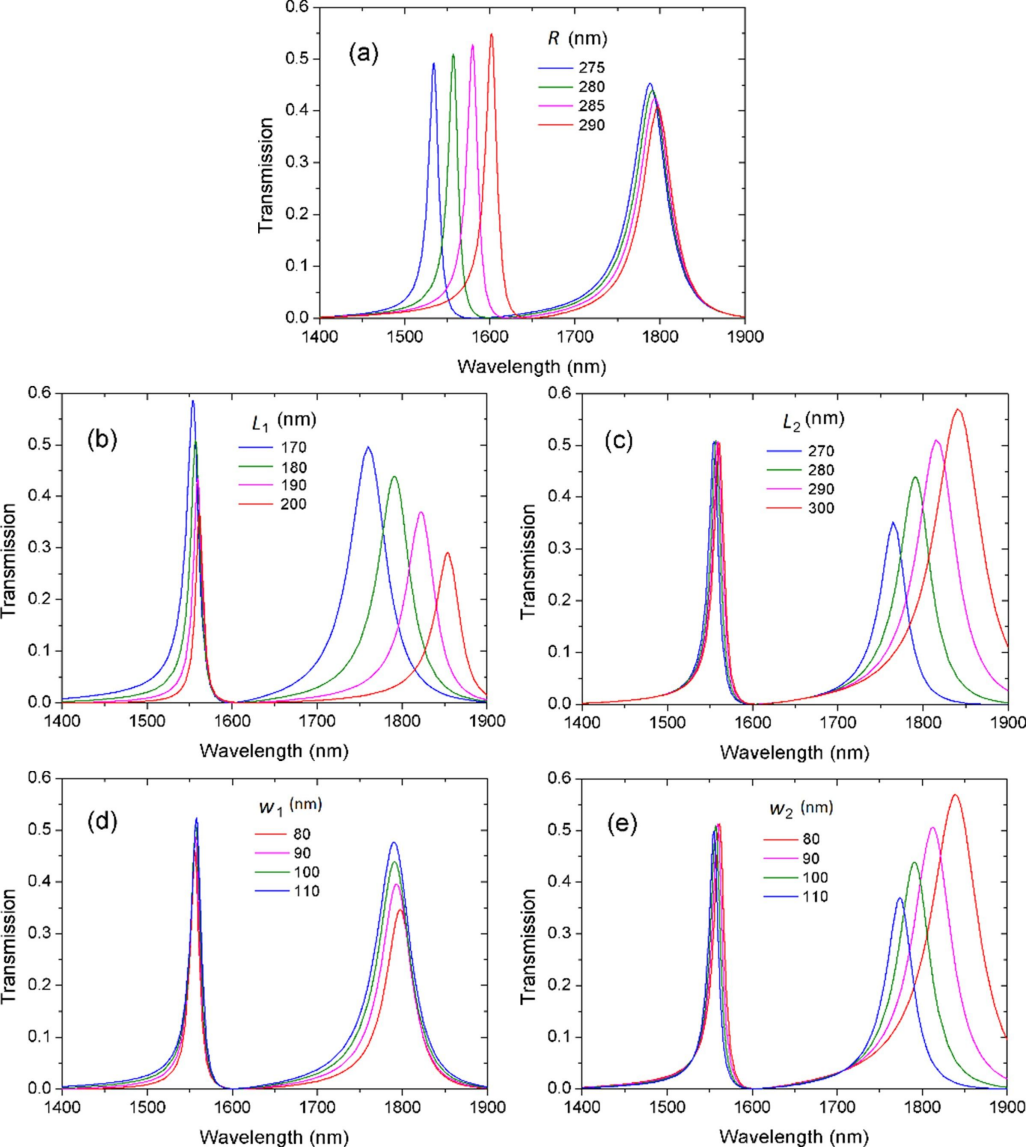
Transmission spectra as functions of the structural parameters: (a) radius of disk *R*; (b) length of upper stub *L*_1_; (c) length of lower stub *L*_2_; (d) width of upper stub *W*_1_; (e) width of lower stub *W*_2_.

In order to verify the above inference, we investigate the transmission when changing the structural size of the resonators. Figure 4a presents the transmission as a function of the disk radius *R*. The spectra at different stub lengths and widths are plotted in Figure 4b–e. The position of transmission peak I exhibits an obvious red shift with the increase of *R* (Figure 4a). In contrast, this peak is hardly affected when the length (*L*_1_, *L*_2_) or the width (*W*_1_, *W*_2_) of the stubs change (Figure 4b–e). The resonance wavelength of peak I is mainly determined by the disk resonator. This indicates that peak I originates from the coupling between disk and stub 1. Figure 4a shows that, in contrast to peak I, the position of transmission peak II remains almost unchanged with the increase of *R*. Nevertheless, peak II is shifted to longer wavelengths when the length (*L*_1_, *L*_2_) of the stubs increases or when the width (*W*_1_, *W*_2_) of stubs decreases (Figure 4b–e). The resonance wavelength of peak II is strongly influenced by the two stubs. This indicates that peak II is related to the stub 1 and stub 2. The simulated results confirm the theoretical prediction.

Figure 4 shows that flexible modulations of the resonance wavelengths can be realized. Two resonance peaks can be easily tuned to certain wavelengths by changing specific structural parameters. This independently tunable dual resonance is promising in the application of multi-parameter sensing.

Next we investigate the effect of the refractive index on the transmission peaks. Since the left resonance peak is mainly influenced by the disk, we fill the disk cavity with a sensing medium, namely a magnetic fluid. The refractive index of the MF (*n*_MF_) varies with temperature *T* and external field strength *H*. The value of *n*_MF_ does not change until the field strength exceeds a critical value *H*_c_. Then, *n*_MF_ increases with rising field strengths and finally reaches a saturation value *n*_sat_. *n*_MF_ follows the Langevin function [2]:

[15] $$\begin{array}{l} n_{\text{MF}}\left(H,T\right)\text{=}\left(n_{\text{sat}}-n_{0}\right)\left[\coth\left(\frac{H-H_{\text{c}}}{T}\right)-\left(\frac{T}{\alpha \left(H-H_{\text{c}}\right)}\right)+n_{0}\right],\\ \text{for }H>H_{\text{c}}. \end{array}$$

In Equation 15, *H* is the magnetic field strength, *T* represents the temperature, and *α* is a fitting parameter. *H*_c_ is the critical field (ca. 30 Oe). *n*_0_ denotes the refractive index of the magnetic fluid at magnetic fields lower than the critical field. It depends on the type of carrier liquid and the concentration of the magnetic fluid. *n*_sat_ is the saturation value of the refractive index. In this paper, the magnetic fluid is a colloidal solution consisting of Fe_3_O_4_ nanoparticles dispersed in water with *n*_0_ = 1.4612. The concentration of the magnetic particles is 1.52% at 24.3 °C. The curve of *n*_MF_ as function of *H* becomes saturated at *H* > 200 Oe. The response of the refractive index to *H* is nonlinear, but in the range of 40–100 Oe, the response shows a good linearity. Therefore field strengths of *H* = 40–100 Oe are considered in this paper*. n*_MF_ increases from 1.4623 to 1.464 when the magnetic field *H* increases from 40 to 100 Oe.

**Figure 5:**
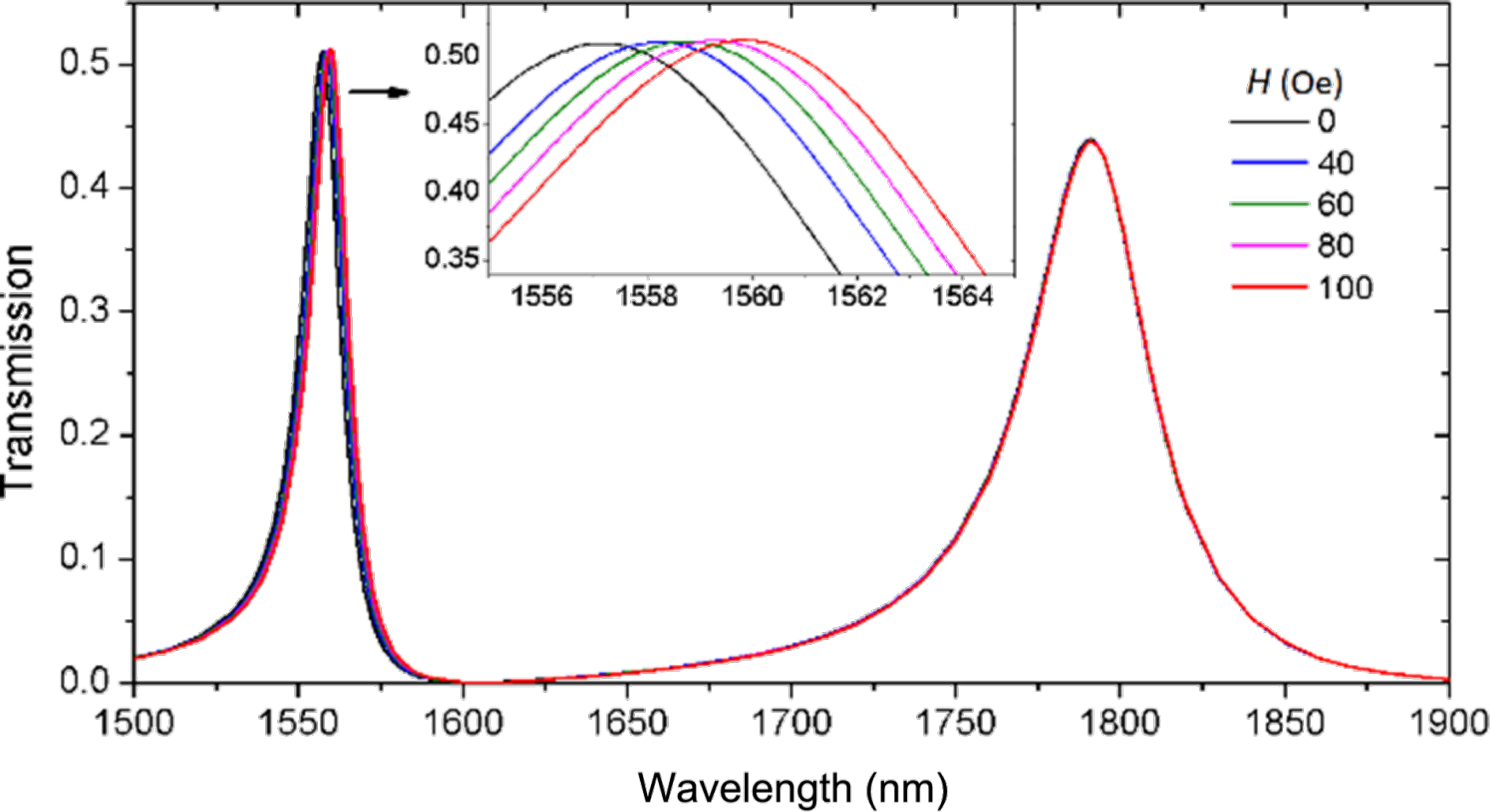
Transmission spectra as a function of the external magnetic field *H*. The parameters are same as in Figure 3 with the exception of the magnetic field strength.

Figure 5 shows the transmission spectra as a function of the external magnetic field *H*. The left resonance at lower wavelengths is more influenced by the magnetic field strength than the right resonance at higher wavelengths. The left resonance is used for detecting and the unchanged resonance serves as a reference signal. The left peak exhibits a red shift with the increase of field strength. Because of the small change of the refractive index, Δ*n*_MF_ = 0.0017, the shift of the left peak is not as large as that in Figure 4. The inset shows an enlarged view of the left peak. The detailed data show that the peak wavelength increases linearly with the magnetic field. Therefore, the magnetic field strength is known by measuring the resonance wavelength shift.

The sensitivity of an refraction-index sensor is usually defined as the shift in the resonance wavelength per unit variation of refractive index (nm/RIU) [34–35]. The obtained sensitivity for the left peak is 946 nm/RIU, which is comparable to other results [36–39]. For a magnetic sensor, the sensitivity is defined as the wavelength shift per unit variation of magnetic field strength (pm/Oe). The sensitivity obtained here is 27 pm/Oe, i.e., 270 pm/mT, which is excellent compared to other reported values [12–15]. Because the right resonance is not sensitive to a change of the external magnetic field, it can be used as a reference. This is the characteristic of a self-reference sensor [37,40]. Thus, a self-reference magnetic -field sensor is achieved and the detection accuracy of the sensor can be improved. Our proposed magnetic sensor can be used in unstable and complicated environments.

## Conclusion

We have demonstrated dual resonances originating from different coupling components. The resonance wavelengths are independently tuned by varying specific structural parameters. By combining a magnetic fluid and a plasmonic structure, a compact sensor that can detect the change of magnetic field strengths is achieved. The sensitivity is as high as 946 nm/RIU for the refraction-index sensor and 27 pm/Oe (or 270 pm/mT) for the magnetic-field sensor, respectively. The sensor features low cost, ease of fabrication and high sensitivity. In particular, because the two resonance peaks exhibit a different dependence on the change in refractive index of the magnetic fluid, the magnetic-field sensor possesses a self-reference characteristic, which can improve the accuracy of sensing. The compact magnetic sensor with high-performance is promising in the area of integrated nanoscale sensing.
